# A transposon mutant library of *Bacillus cereus* ATCC 10987 reveals novel genes required for biofilm formation and implicates motility as an important factor for pellicle‐biofilm formation

**DOI:** 10.1002/mbo3.552

**Published:** 2017-11-22

**Authors:** Mira Okshevsky, Matilde Greve Louw, Elena Otero Lamela, Martin Nilsson, Tim Tolker‐Nielsen, Rikke Louise Meyer

**Affiliations:** ^1^ Interdisciplinary Nanoscience Center Aarhus University Aarhus Denmark; ^2^ Department of Immunology and Microbiology University of Copenhagen Copenhagen Denmark; ^3^ Department of Bioscience Aarhus University Aarhus Denmark

**Keywords:** *Bacillus cereus*, biofilm, biofilm extracellular matrix, *dlt* operon, motility

## Abstract

*Bacillus cereus* is one of the most common opportunistic pathogens causing foodborne illness, as well as a common source of contamination in the dairy industry. *B. cereus* can form robust biofilms on food processing surfaces, resulting in food contamination due to shedding of cells and spores. Despite the medical and industrial relevance of this species, the genetic basis of biofilm formation in *B. cereus* is not well studied. In order to identify genes required for biofilm formation in this bacterium, we created a library of 5000 +  transposon mutants of the biofilm‐forming strain *B. cereus* ATCC 10987, using an unbiased mariner transposon approach. The mutant library was screened for the ability to form a pellicle biofilm at the air‐media interface, as well as a submerged biofilm at the solid‐media interface. A total of 91 genes were identified as essential for biofilm formation. These genes encode functions such as chemotaxis, amino acid metabolism and cellular repair mechanisms, and include numerous genes not previously known to be required for biofilm formation. Although the majority of disrupted genes are not directly responsible for motility, further investigations revealed that the vast majority of the biofilm‐deficient mutants were also motility impaired. This observation implicates motility as a pivotal factor in the formation of a biofilm by *B. cereus*. These results expand our knowledge of the fundamental molecular mechanisms of biofilm formation by *B. cereus*.

## 1 INTRODUCTION

*Bacillus cereus* is a gram‐positive spore‐forming bacterium found in soil and other natural environments. It causes diarrheal and emetic food poisoning in humans (Kotiranta, Lounatmaa, & Haapasalo, 2000), and is a common source of contamination in the dairy industry (Gopal et al., 2015; Kalogridou‐Vassiliadou, 1992). The ability of *B. cereus* to form robust biofilms that are recalcitrant to cleaning and disinfection is a major reason for its success as a dairy‐contaminant (Brooks & Flint, 2008; Kumar & Anand, 1998). *Bacillus* species form biofilms at the solid‐liquid interface, referred to here as submerged biofilms, as well as pellicle biofilms at the liquid‐air interface. Cells and spores detach from these biofilms and lead to food contamination, and potentially also to food poisoning. Despite the medical and industrial relevance of this ubiquitous bacterium, the genetic basis of biofilm formation in *B. cereus*, especially compared to its well‐studied relative *Bacillus subtilis*, is not well understood.

In the plant‐associated bacterium *B. subtilis*, the switch from planktonic to biofilm mode proceeds via well characterized genetic pathways. For a comprehensive review see (Vlamakis, Chai, Beauregard, Losick, & Kolter, 2013). Briefly stated, environmental signals are sensed by histidine kinases A–D, which activate a phosphorelay cascade (Jiang, Shao, Perego, & Hoch, 2000). Phosphorylation of the master transcriptional regulator Spo0A results in activation of biofilm‐associated genes at low levels of phosphorylation (Fujita, González‐Pastor, & Losick, 2005). Spo0A‐P upregulates the antirepressor SinI, which derepresses the dedicated biofilm repressor SinR (Lewis, Brannigana, Smith, & Wilkinson, 1996). Derepression of SinR allows the biofilm‐matrix‐encoding operons *epsA‐O* and *tapA‐sipW‐tasA* to be transcribed. TasA is a protein monomer which assembles into long amyloid‐like fibers after having been processed by the signal peptidase SipW. This protein and the exopolysaccharide encoded by the *espA‐O* operon are the most important components of the biofilm extracellular matrix in *B. subtilis* (Branda, Chu, Kearns, Losick, & Kolter, 2006; Branda et al., 2006). Additional genes known to play a role in *B. subtilis* biofilm formation are *bslA*, which confers hydrophobicity to the biofilm surface (Kobayashi & Iwano, 2012), and the *degS/degU* sensor relay system (Kobayashi, 2007; Murray, Kiley, & Stanley‐Wall, 2016).

A pellicle is a floating biofilm formed at the air‐liquid interface. Formation of a pellicle begins with the differentiation of single motile cells into cell chains, or filaments, which subsequently bundle together with the aid of extracellular polymeric substances. Functional regulators *sigH*,* slr*, and *spo0A* are required for the formation of these cell chains in *B. subtilis* (Kobayashi, 2007), and loss of either *eps* or *tasA* biofilm matrix components abolishes cell bundling (Branda et al., 2006; Kobayashi, 2007). Regulators *abh*,* alsR*,* sigX*,* yvrH*, and *cysL* are additionally required for cell chains to form bundles (Kobayashi, 2007). Pellicle morphology of *B. cereus*, however, is distinct from that of *B. subtilis*. While *B. subtilis* forms pellicles with a wrinkled architecture that do not adhere to the sides of culture vessels (Kobayashi, 2007), *B. cereus* pellicles adhere strongly to the sides of culture vessels, and form flat pellicles without folds or wrinkles (Fagerlund et al., 2014). These different pellicle morphologies suggest that the underlying molecular mechanisms of pellicle formation in these two species differ.

Several distinct differences between the genetic regulation of biofilm formation in *B. cereus* and *B. subtilis* have already been observed experimentally. *B. cereus*, for example, contains two homologues of *tasA* but no homologues of *tapA* (Caro‐Astorga, Pérez‐García, de Vicente, & Romero, 2015). The two‐component regulatory system DegU/DegS is not present in *B. cereus* (Ivanova et al., 2003), while the *plcR* virulence regulator, responsible for modulating the expression of 45 genes in *B. cereus*, is completely absent in *B. subtilis* (Gohar et al., 2008). Due to the genetic heterogeneity between the two species, we decided to clarify the genetic basis of biofilm formation in *B. cereus* by conducting a genome‐wide screening of genes required for both pellicle‐biofilm and submerged‐biofilm formation.

The *himar1* transposon is known to insert into genomes of gram‐positive bacteria at random (Cao, Bitar, & Marquis, 2007), thereby inactivating genes in an unbiased manner. In order to maximize the chances of identifying novel genes for biofilm formation, we chose to investigate *B. cereus* strain 10987, which is especially good at biofilm formation when compared with *B. cereus* type strain ATCC 14579 (Auger, Krin, Aymerich, & Gohar, 2006; Hsueh, Somers, Lereclus, & Wong, 2006). Several unique characteristics of this strain may account for this increased biofilm forming ability. Strain ATCC 10987 biofilm cells, for example, have been shown to have a lower protein:carbohydrate ratio than the type strain, a completely different extracellular proteome, and a more hydrophobic surface (Karunakaran & Biggs, 2010). The explanation for these unique characteristics and excellent biofilm forming ability of *B. cereus* 10987 lies in its genome, which is fully sequenced (GenBank accession number AE017194.1). Molecular investigations into strain ATCC 10987 have previously been hindered by this strain's extensive system of restriction endonucleases, which makes the establishment of a genetic model system very difficult. Byusing recently developed “mimicking‐of‐DNA‐methylation‐patterns” pipeline (Zhang et al., 2014), we were able to transform *B. cereus* 10987 and produce a library of 5,019 transposon mutants.

In order to identify novel genes required for biofilm formation, we screened this library for the ability to form pellicle and submerged biofilms. These screenings revealed 88 genes required for pellicle biofilm formation, as well as six genes required for submerged biofilm formation at the solid‐media interface. Furthermore, investigations suggested that many mutants were deficient in motility, and swimming assays were therefore conducted on all mutants.

## 2 METHODS

### 2.1 Bacterial strains and media

The strains used in this study were *B. cereus* ATCC 10987, *Escherichia coli* DH5α harbouring the pBTn plasmid*,* and *E. coli* EC135 harboring the pM.Bce plasmid with the *B. cereus* 10987 methylation genes. Bacteria were stored in glycerol stock at −80°C. They were streaked onto 15 g/L LB agar (LB, Luria–Bertani Millers, Fluka, pH 7.0) prior to a new experiment. Antibiotics were added to the media when appropriate in the following concentrations: chloramphenicol 10 μg/ml, erythromycin 5 μg/ml, spectinomycin 20 μg/ml, and ampicillin 100 μg/ml.

### 2.2 Transformation of *Bacillus cereus* 10987 with pBTn

Plasmid pBTn (ca. 400 ng) was mixed with 100 μl electrocompetent *E. coli* pM.Bce (Zhang et al., 2014) cells and electroporated with a Biorad Electroporator at 25 μF 600 Ω and 1.8 kV/mm. Immediately following electroporation, 500 μl SOC media was added and mixture was incubated for 1 hr at 37°C. Chloremphenicol and spectinomycin were then added and cells were incubated further for 50 min at 37°C before being spread on LB agar + chloramphenicol + spectinomycin or on LB agar + spectinomycin + ampicillin. Plates were incubated overnight at 30°C. *In vivo* methylation of pBTn was performed as described in Zhang et al. (2012). Briefly, one colony of *E. coli* pBTn pM.Bce was inoculated into LB broth with erythromycin, spectinomycin, chloramphenicol, and ampicillin to ensure selection of both plasmids, and with 0.2% (w/vol) L‐arabinose to induce transcription of methylase genes. Cells were incubated at 30°C 180 rpm overnight. Plasmids were extracted with plasmid extraction kit Gene Elute™ (Sigma) and plasmid solution was micro‐dialysed by placing the solution on a 0.025 μm filter (Millipore) floating on dH_2_O for 2 hr. Two μg of plasmid was mixed with 70 μl competent *B. cereus* 10987 cells and electroporated at 25 μF 400 Ω 1.25 kV/mm. One ml SOC media was added and incubated for 30 min at 37°C. Chloramphenicol was then added and cells incubated a further 30 min at 37°C before being spread on LB agar + chloramphenicol and incubated at 30°C until colonies formed. Colonies were then transferred to LB agar + erythromycin to confirm transformation. Transformation of *B. cereus* ATCC 10987 was confirmed with PCR using primers IRXba and IRPst (Li, Shibata, Takeshita, & Yamashita, 2014) on plasmid extracted from *B. cereus* 10987 pBTn, with plasmid extractions from *E. coli* pBTn serving as positive control, and genomic DNA extracted from *B. cereus* 10987 serving as a negative control to exclude genomic targets for the primers. PCR products were verified on a 1.5% agarose gel.

### 2.3 Production of *Bacillus cereus* 10987 transposon mutant library

*Bacillus cereus* 10987 pBTn was grown overnight in LB + chloramphenicol + erythromycin at 30°C and 180 rpm. The culture was diluted 1:100 in LB + erythromycin + 0.5% xylose to induce transposase transcription and grown at 30°C, 180 rpm overnight. The cultures were then diluted 1:100 in LB + eryt hromycin + 0.5% xylose and incubated at 43°C to rid cells of the plasmid. This step was repeated once for a total of 2 days incubation before cells were spread on LB agar + erythromycin and grown overnight at 30°C. This entire procedure was repeated five times in order to increase the number of mutants obtained. Resulting colonies were transferred to LB agar + chloramphenicol and LB agar + erythromycin and incubated overnight at 30°C. In total, 5,019 colonies were determined to have the appropriate chloramphenicol sensitive/erythromycin resistant phenotype, and were inoculated into LB + erythromycin for overnight grown before glycerol was added and mutants were stored at **−**80°C.

### 2.4 Screen for pellicle‐deficient mutants

Pre‐cultures grown overnight in LB at 37°C, 180 rpm, were diluted 1:1000 and 200 μl inoculated into the wells of 96‐well plate (Sarstedt), which were sealed into zip‐lock bags prior to incubation at 37°C for 72 hr. Each plate contained three replicate wells of the WT to serve as positive control, and three wells containing only media to serve as negative control. Pellicles were evaluated on a +/− basis, with mutants either able or unable to form a pellicle visually determined to be similar to the WT. This assay was repeated three times, with those mutants determined to be pellicle negative (−) failing to form a pellicle in all replicates and in all three experiments.

### 2.5 Screen for submerged‐biofilm deficient mutants

A high‐throughput microtiter‐assay was performed to identify conditions under which wild type *B. cereus* 10987 reliably produced submerged biofilms on the solid‐media interface. Based on these results (not shown), TSB + 0.5% yeast extract was determined to be optimal media for submerged biofilm screening. Wild type *B. cereus* 10987 and all mutants were grown overnight in LB at 37°C, 180 rpm, before 160 μl of overnight culture was added to wells of a 96‐well plate (Nunc). A peg‐lid (Nunc Immuno TSP lid) was then immersed in this pre‐culture for 1 hr at 37°C for inoculation before being moved to a fresh 96‐well plate containing 160 μl of TSB + 0.5% yeast extract in all wells. Plates were placed into a zip‐lock bag to reduce evaporation, and incubated at 37°C for 24 hr. Peg lids were then removed and placed upside down to air dry for 20 min. OD_600_ of all wells was measured to confirm growth. Peg lids were immersed in a 96‐well plate containing 180 μl/well 0.5% crystal violet (Sigma) for 15 min, before being rinsed three times in dH_2_O to remove unbound stain. Crystal violet was extracted from biofilms in 180 μl/well 96% ethanol for 15 min, and OD_585_ of the extracted stain was measured in a plate reader (BioTek Power wave XS2). Biofilm‐defective mutants were defined as those mutants that showed no significant difference from the negative control (blank media), in all replicates in all three repetitions of the screening assay.

### 2.6 Arbitrary PCR

To determine the disrupted genes in the biofilm‐deficient mutants, the arbitrary PCR method was used to determine the nucleotide sequences flanking the transposon insertion sites. Genomic DNA (gDNA) was extracted using GeneJet Genomic DNA Purification kit from Thermo Scientific as per manufacturer's instructions. PCR was performed using Red Taq PCR mixture (Sigma‐Aldrich) and MgCl_2_ (50 mmol/L). The first PCR was performed on template gDNA extracted from the mutants with the primers Arb 2 and Erm 5.3 (Table 1) at a final volume of 50 μl. The PCR was performed as follows: 95°C for 5 min, 30x (95°C for 30 s, 38 °C for 30 s and 72°C for 2 min), and 72°C for 5 min. Purified PCR products were used as template for the second PCR using primers Arb 3 and Erm 5.1 (Table 1) at a final volume of 100 μl. The PCR was performed as follows: 95°C for 5 min, 30x (95 °C for 30 s, 55 °C for 30 s, and 72°C for 2 min), and 72°C for 5 min. All plasmids and primers used in this study are described in Table 1.

**Table 1:** Plasmids and primers used in this study

Plasmid/primer	Description	Reference
pBTn	Temperature sensitive replication system from pBT2 (Brückner, 1997), chloramphenicol resistance gene, erythromycin resistance gene (*ermB*) between himar1 transposon inverted repeats, himar1 mariner transposase under control of xylose inducible promotor	Li et al. (2014)
pM.Bce	Spectinomycin resistance, six *Bacillus cereus* specific methylases under control of arabinose inducible promotor	Zhang et al. (2014)
pMK3	A first‐generation shuttle vector capable of replication in *Escherichia coli* and *Bacillus*. Confers ampicillin resistance in *E. coli* and Kanamycin resistance in *Bacillus*. Allows blue/white screening in *E. coli*	Sullivan, Yasbin, and Young (1984)
IRXba	TCTGTCCGAGAGTGATTGGTCTTGCGTATGG	Li et al. (2014)
IRPst	GGTTGGCTGATAAGTCCCCGGTCT	
Arb2	GGCCACGCGTCGACTAGTCANNNNNNNNNNGATCA	
Arb3	GGCCACGCGTCGACTAGTCA	
Erm5.1	GCTTCTAAGTCTTATTTCCATAAC	
Erm5.3	TCTACATTACGCATTTGGAATAC	
dra forward	CATTGGATCCGCGGACCATTGGAATTGAGT	This study
dra reverse	CATTGGATCCTAAAGTCGCGCTCACCTT	
dltB forward	CATTGGATCCTCGTGCGTTTTCCGGGAA	
dltB reverse	CATTGGATCCTGCCCACATGCATGCATT	

Restriction sites used for cloning are underlined.

### 2.7 Identification of transposon insertion sites and disrupted genes

Products obtained from the arbitrary PCR method were purified using Gen Elute PCR Clean‐UP kit from Sigma as directed by the manufacturer. Purified samples were diluted in PCR‐grade water to a concentration of approx. 25 ng/ul and sequenced using Sanger sequencing (Macrogen Inc., South Korea) with primer Erm 5.1. Resulting sequencing quality was assessed based on the chromatogram and sequences were trimmed manually. Geneious version R7 (Kearse et al., 2012) was used to map sequencing reads to the *B. cereus* 10987 reference genome (GenBank accession number AE017194, version AE017194.1) using the parameters “highest sensity/slow,” and “iterate up to five times.” A multiple sequence alignment was first performed on the sequencing reads using Geneious vR7 to determine the identical region corresponding to the transposon itself, which allowed us to determine the exact location of transposon insertion (i.e.,, the first base belonging to the genome following the transposon sequence “GTTA”). Genes annotated by GenBank as “putative protein” were additionally investigated using KEGG and Prosite databases, to gain insight into potentially functional protein domains.

### 2.8 Complementation of selected biofilm‐defective mutants

Genomic DNA was extracted from *B. cereus* 10987 using GeneJet genomic DNA extraction kit from Sigma. Genes of interest were amplified from the wildtype genome using primers listed in Table S1 The resulting PCR fragments were gel purified using GenEluteTM Gel Extraction Kit (Sigma), digested with FastDigest^®^ enzymes obtained from ThermoFischer, and ligated into the multiple cloning site of shuttle vector pMK3 (obtained from the Bacillus Genetic Stock Center) at either BamHI or SalI, using T4 DNA ligase (ThermoFischer). The resulting ligate was transformed into OneShot chemically competent TOP10 *E. coli* (ThermoFischer) using heat shock, and blue/white screening was employed on LB agar + ampicillin + Xgal to identify colonies harboring plasmids with insertions. Plasmids were extracted using GeneJet plasmid extraction kit and proper insertion determined by sequencing. Plasmids harboring correct insertions were transformed into *E. coli* pM.Bce (Zhang et al., 2014) using electroporation as described above, in order to obtain a *B. cereus*‐type methylation pattern on the plasmid. *E. coli* pM.Bce pMK3‐*geneX* was grown overnight in the presence of 0.4% DL‐arabinose to induce methylation. The methylated complementation vectors were extracted using GeneJet plasmid extraction kit and transformed into the appropriate *B. cereus* transposon mutant via electroporation as described above. Transformants were selected on LB agar + kanamycin (pMK3 confers kanamycin resistance in *Bacillus*) + erythromycin (resistance conferred by transposon insertion).

### 2.9 Motility assay

Overnight cultures were grown in LB at 37°C, 180 rpm. Five uL of bacterial culture was stabbed into the center of LB‐ agar plates containing 0.2% agar, and the plates incubated at room temperature for 24 hr prior to observation. The assay was evaluated on a yes/no basis, with mutants either able (yes) or unable (no) to form a swimming halo.

### 2.10 Statistical analysis

Biofilm biomass of the mutants as determined by crystal violet staining (described above) was compared to the wild type biomass using student *t*test. A *p*‐value of ≤.05 was considered significant. All experiments claiming statistical significance were conducted with at least three biological replicates and three technical replicates.

## 3 RESULTS AND DISCUSSION

### 3.1 Identification of genes required for biofilm formation

**Figure 1:**
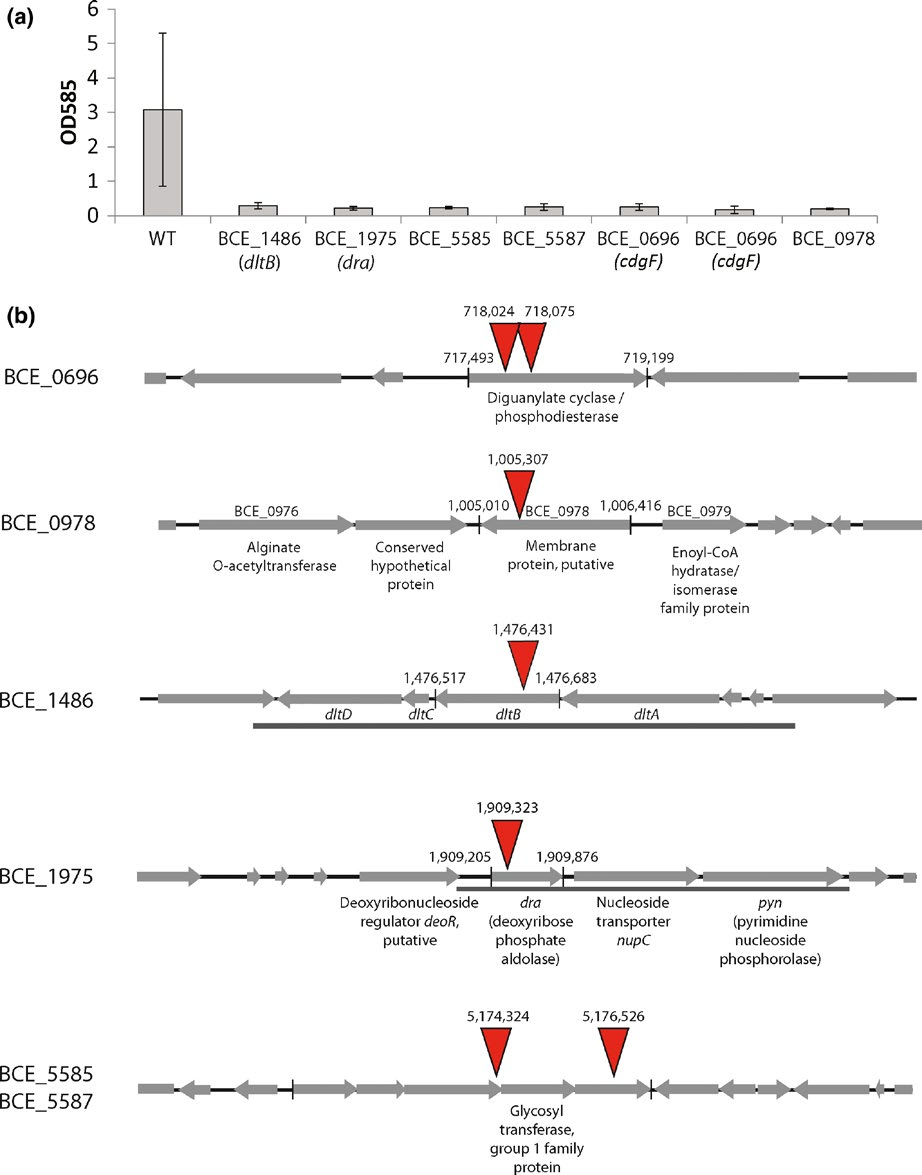
Transposon insertions resulting in mutants deficient in submerged‐biofilm formation. (a) Quantification of biofilm biomass by crystal violet assay, (b) location of transposon insertions in genome indicated by red arrow. Grey bars below genes show regions that were genetically complimented in *dra* (BCE_1975) and *dltB* (BCE_1486) mutants

Following transformation of *B. cereus* ATCC 10987 with plasmid pBTn, we produced 5,019 mutants containing a random *hrm1* transposon insertion in the genome. These mutants were screened for the inability to form a pellicle biofilm at the air‐media interface after 3 days of incubation in microtiter plates, and were also screened for the ability to form submerged biofilm on the surface of polystyrene pegs. Those mutants which grew comparably to the wild type in planktonic culture, but were unable to form a pellicle after 3 days, were determined to be pellicle deficient. Those mutants which grew comparably to the wild type in planktonic culture, but were unable to attach to the peg surface after 24 hr, were determined to be submerged‐biofilm deficient. The DNA sequence of the region flanking the transposon insertion site was successfully obtained from 115 unique pellicle‐deficient mutants (Table 2 and Table S1), and seven unique submerged‐biofilm‐deficient mutants (Table 3). Of the six genes identified as necessary for submerged‐biofilm formation, three were also identified in the pellicle screening. Genes BCE_5585 and BCE_5587, members of a novel biofilm‐associated operon, and BCE_0978 were the only genes identified in the submerged biofilm screening that were not also identified in the pellicle screening (Table 3 and Figure 1). The total of 119 mutants represent 91 genes putatively required for biofilm formation by *B. cereus* ATCC 10987.

**Table 2:** Selected genes identified in this study to be essential for pellicle‐biofilm formation by *Bacillus cereus* (full list of 91 genes in Table S1)

Gene locus tag	Number of independent transposon insertions	Location of transposon insertion (base pair number)	Gene name/annotation	Predicted function	Pathway/Functional group	Swim (yes/no)
*Amino acid metabolism*						
BCE_1486	2	1,475,690; 1,476,433	*dltB*	Membrane protein involved in export of D‐alanine	Cationic antimicrobial peptide (CAMP) resistance; two component systems;	N/A
BCE_1487	2	1,477,422; 1,477,539	*dltA*	D‐alanine activating enzyme/D‐alanine poly(phosphoribitol) ligase subunit	D‐Alanine metabolism; cationic antimicrobial peptide (CAMP) resistance; two component systems	No
BCE_4209 (upstream)	1	3,925,781	*sdhB*	L‐serine dehydratase, iron‐sulfur dependent, beta subunit	Glycine, serine and threonine metabolism; cysteine and methionine metabolism; biosynthesis of amino acids	Yes
BCE_4712	1	4,365,352	*speD*	s‐andenosylmethionine decarboxylase proenzyme; catalyzes the decarboxylation of S‐adenosyl methionine to S‐adenosyl methioninamine	Cystein and methionine metabolism; Arginine and proline metabolism; amino acid metabolism (Methionine salvage pathway; Polyamine biosynthesis, arginine ‐> agmatine ‐> putriscine ‐> spermidine	No
*Cell growth and division*						
BCE_5742	1	32,104	Bc23SB	23S ribosomal RNA	Ribosome biogenesis	No
BCE_5745	1	84,328	Bc23SC	23S ribosomal RNA	Ribosome biogenesis	No
BCE_0097 (upstream)	1	104,890	ribosomal protein L11		Ribosome biogenesis	No
BCE_5763	1	335,253	Bc23S1	23S ribosomal RNA	Ribosome biogenesis	Yes
BCE_4353	1	4,038,796	cell elongation‐specific peptidoglycan D,D‐transpeptidase		Cell elongation	No
BCE_5301	1	4,887,884	*lytE*	Endopeptidase; cell wall hydrolyses	Cell division	No
BCE_5633	1	5,218,114	*gidB*	16S rRNA (guanine527‐N7)‐methyltransferase	Ribosome biogenesis/modification	N/A
BCE_5634	2	5,219,259; 5,219,518	*gidA*	tRNA uridine 5‐carboxymethylaminomethyl modification enzyme	Chromosome partitioning	No
*Flagellar motility*						
BCE_1756	1	1,710,069	flagellar M‐ring protein		Flagellar assembly/bacterial chemotaxis	No
BCE_1767	1	1,710,735	*fliG*	Flagellar motor switch protein	Flagellar assembly/bacterial chemotaxis	No
BCE_1780	2	1,722,347; 1,722,507	*fliC*	Flagellin	Flagellar assembly/bacterial chemotaxis	No
DNA replication/modification						
BCE_5617	1	5,204,841	*purA*	Adenylosuccinate synthetase; adenine ribonucleotide biosynthesis	Purine metabolism; alanine, aspartate and glutamate metabolism	No
RNA modification/transcription/regulation						
BCE_0875	3	901,507; 901,619; 901,882	transcription antiterminator, LytR family		Transcriptional regulator	No
BCE_1415	1	1,418,392	*pdhR*	Transcriptional regulator, GntR family, represses pyruvate dehydrogenase complex	Transcriptional regulator	No
BCE_4150	2	3,874,856; 3,875,001	transcriptional regulator marR family		Transcriptional regulator	No
BCE_5280	1	4,870,304	transcriptional regulator, LysR family		Transcriptional regulator	N/A
BCE_5481	2	5,060,626; 5,060,653	*plcR*, putative	Transcriptional regulator	Transcriptional regulator	N/A
BCE_5613	1	5,200,504	YycH	Regulatory protein	Regulation/signal transduction	No
*Sporulation*						
BCE_4540	1	4,208,761	*minD*	Septum‐site determining protein MinD	Sporulation related gene, comes right before spoIVFA, chromosome partitioning protein, inhibitor of FTSZ assembly	No
BCE_5636	1	5,222,600	*jag*	spoIIIJ‐associated protein	Sporulation	No
*Sugar metabolism*						
BCE_1975	1	1,909,330	*deoC*/*dra*	Deoxyribose phosphate aldolase	Pentose phosphate pathway	No
BCE_5356	1	4,943,951; 4,943,954	glycosyl transferase domain protein, putative		Carbohydrate/sugar metabolism	No
BCE_5385	1	4,972,962	*ugd*	UDP‐glucose 6‐dehydrogenase	Starch and sucrose metabolism; (ascorbate biosynthesis, glucose‐1P ‐> ascorbate; Glucuronate pathway (uronate pathway); Nucleotide sugar biosynthesis	No
BCE_5386	1	4,974,317	polysaccharide transport protein, putative		Polysaccharide transporter	No
BCE_5398	1	4,987,306	capsular exopolysacchraide family protein		Exopolysaccharide production	No
BCE_5586	1	5,175,279	glycosyl transferase, group 1 family protein		Carbohydrate/sugar metabolism	N/A
BCE_5588 (downstream)	1	5,177,285	*galE*	Catalizes conversion of galactose to alpha‐D‐glucose	Galactose metabolism; amino sugar and nucleotide sugar metabolism (nucleotide sugar biosynthesis; galactose degradation,)	No
*Transporters*						
BCE_0140	1	136,978	ABC transporter, ATP‐binding protein		Transporter	No
BCE_0245	1	247,451	ABC transporter, ATP‐binding protein		Transporter	No
BCE_1840	1	1,777,050	Na+/H+ antiporter, NhaC family		Transporter	N/A
BCE_3309	1	3,099,433	Major facilitator family transporter		Transporter	No
BCE_3917	1	3,665,382	cation‐transporting ATPase, E1‐E2 family		Transporter	No
BCE_5200	1	4,795,500	sodium/alanine symporter family protein		Transporter	No
*Other*						
BCE_0696	1	718,076	sensory box/GGDEF family protein	Diguanylate cyclase; converts two GTP to cyclic di‐GMP	Nucleotide metabolism; virulence signaling	No
BCE_3314	1	3,104,049	*slo*	Thiol‐activated cytolysin	Quorum sensing; Nod‐like receptor signaling pathway	No
BCE_5571	1	5,158,425	antiholin‐like protein LrgB		Two‐componant system (signal transduction)	No

**Table 3:** Summary of genes identified in this study to be necessary for *Bacillus cereus* to form submerged biofilms at the solid‐liquid interface

Gene	Gene name	Gene annotation	Function	Location of gene (bp)	Transposon insertion location (bp)	Also identified in pellicle screening?	Swim (yes/no)
BCE_0696	*cdgF*	sensory box/GGDEF family protein	Diguanylate cyclase (produces cyclic‐di‐GMP)	717,493–719,199	718,075	Yes	No
BCE_0696					718,024		
BCE_0978	—	membrane protein, putative	Unknown	1,005,010–1,006,416	1,005,307	No	No
BCE_1486	*dltB*	dltB protein	Part of a four‐gene operon for D‐alanyl‐lipoteichoic acid biosynthesis. Possibly involved in transport of d‐alanine over the membrane	1,476,683–1,475,517	1,476,431	Yes	No
BCE_1975	*dra*	Deoxyribose phosphate aldolase	Catalyzes the formation of D‐glyceraldehyde 3‐phosphate and acetaldehyde from 2‐deoxy‐D‐ribose 5‐phosphate in nucleotide catabolism	1,909,205–1,909,876	1,909,323	Yes	No
BCE_5585	—	Membrane protein, putative	Unknown. Pfam notes “uncharacterised protein conserved in bacteria”	5,172,537–5,174,369	5,174,324	No	Yes
BCE_5587	—	Membrane protein, putative	Unknown. Pfam predicts a putative exopolysaccharide exporter	5,175,782–5,177,236	5,176,526	No	Yes

**Figure 2:**
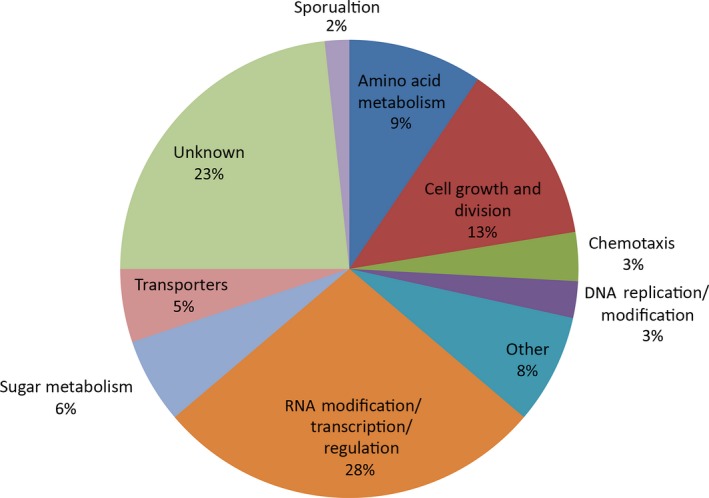
Functional categorization of unique transposon insertions resulting in pellicle‐negative mutant phenotypes

The 115 transposon insertions resulting in pellicle‐deficient phenotypes were grouped into categories based on the function of the disrupted gene. GenBank annotations and the Kyoto Encyclopedia of Genes and Genomics (KEGG) were used to identify putative gene functions on the basis of sequence homology. The functional categories that the 115 insertions fell into are; amino acid metabolism; cell growth, division and respiration; flagellar motility; DNA replication/modification; RNA modification/transcription/regulation; sporulation; sugar metabolism; transporters; other functions; and unknown functions. The relative distribution of the 115 unique transposon insertions within these functional categories is shown in Figure 2.

### 3.2 Genes involved in amino acid metabolism

Eleven transposon insertions (9% of total) resulting in a pellicle‐negative phenotype occurred in genes involved in amino acid metabolism. For example, **S**‐andenosylmethionine decarboxylase proenzyme (*speD*) catalyzes the decarboxylation of S‐adenosylmethionine to S‐adenosylmethioninamine, which is the propylamine donor required for the synthesis of the polyamines spermine and spermidine from the diamine putrescine. Polyamines have been identified as important factors for biofilm formation in other bacteria such as *Vibrio cholera* (Karatan, Duncan, & Watnick, 2005), *Neiserria gonoerrehae* (Goytia, Dhulipala, & Shafer, 2013), and *B. subtilis* (Cao et al., 2012; Hobley et al., 2014). No link between spermine and/or spermidine and biofilm formation by *B. cereus* has as of yet been experimentally determined. Because the speD mutant was also motility impaired, biofilm formation may be hampered solely due to the mutants inability to swim to the surface. The identification of this mutant as biofilm‐deficient suggests that a link between polyamine synthesis and biofilm formation exists in *B. cereus*, and calls for further investigation.

L‐serine dehydratase (locus tag BCE_4209) is an iron‐sulfur protein that converts l‐serine to pyruvate and ammonia. *Campylobacter jejuni* mutants of L‐serine dehyratase have been shown to be virulence impaired in chickens (Velayudhan, Jones, Barrow, & Kelly, 2004), which the authors attributed to the crucial nature of L‐serine for *in vivo* growth. This was one of the few pellicle‐deficient mutants that retained motility.

Two mutants in the category of amino acid metabolism harbored transposon insertions in the *dlt*‐operon, which encodes for d‐alanylation of lipoteichoic acids in the cell wall (Perego et al., 1995). The esterification of lipoteichoic acids with positively charged D‐alanyl groups alters the net surface charge and the potential for interaction with cations (Neuhaus & Baddiley, 2003), thereby increasing resistance to cationic antimicrobials (Kovács et al., 2006; Saar‐Dover et al., 2012). The *dlt*‐operon has been experimentally demonstrated to be necessary for biofilm formation in species such as *Enterococcus faecalis* (Fabretti et al., 2006) and *Staphylococcus aureus* (Gross, Cramton, Götz, & Peschel, 2001), although distruption of these genes in *Streptococcus mutans* was shown not to impact biofilm formation (Nilsson et al., 2016). In *B. cereus*, the *dlt*‐operon has been shown to be required for virulence, and resistance to cationic antimicrobials (Khattar et al., 2009), but its role in biofilm formation has not been previously determined. The biofilm‐negative phenotypes of our *dltA* and *dltB* transposon mutants is therefore a novel observation, potentially linked to the mutants inability to swim. However, because the *dlt*‐operon effects biofilm formation in other, naturally non‐motile species, it is likely that the loss of d‐alanylated teichoic acids has more direct effects on the ability of *B. cereus* to form a biofilm. The inability of these mutants may explain why the *dlt*‐operon has been linked to virulence in other studies (Khattar et al., 2009), as lack of motility will directly impact the ability of a bacterium to collonize a host. We have confirmed that our *dltB* transposon mutant is more susceptible than the wild type to cationic antimicrobials (data not shown), which is consistent with what is known from other gram positive species.

### 3.3 Genes for cell growth, division, and respiration

Fifteen independent transposon insertions (13% of total) implicated 13 genes for cell growth, division and/or respiration as essential for pellicle‐biofilm formation. These insertions disrupted genes for ribosome biogenesis and modification, such as the 23S ribosomal RNA subunits A, B, and C, and ribosomal protein L11. Gene *gidA* is a glucose‐inhibited‐division protein, responsible for the modification of tRNA uridine 5‐carboxymethylaminomethyl and one half of the *gibAB* operon. Interestingly, a proteomic analysis of the biofilm and planktonic growth modes of *Acinetobacter baumannii* identified *gidA* as a protein present only in the biofilm growth mode (Shin, Lee, Kim, & Kim, 2009), and *gidA* has been shown to play a regulatory role in the virulence of *Aeromonas hydrophila* (Sha et al., 2004). We have obtained two independent transposon insertions in the *gidA* gene during this study, thereby confirming its importance for pellicle‐biofilm formation, and we additionally observed that *gidA* is required for motility. This deficiency in motility might at least partially explain the relationship between *gidA* and virulence observed in other studies. In our study, a transposon insertion in the *gibB* gene also resulted in a pellicle‐negative phenotype. The role of the *gibAB* operon in virulence and stress response has been recently reviewed (Shippy & Fadl, 2015), and deletion mutants of these genes have been shown to have a deleterious effect on biofilm formation in *Pseudomonas fluorescens* (Zhang et al., 2014), and *Streptococcus mutans* (Li et al., 2014). We contribute the observation that this operon is also important for biofilm formation in *B. cereus*.

Additional genes required for cell division include *lytE*, an endopeptidase involved in cell wall hydrolyses, and gene BCE_4353, a cell elongation‐specific peptidoglycan D,D‐transpeptidase. As cell morphology is an important factor in proper cell bundling, defects in cell wall elongation or separation could be responsible for the biofilm‐negative phenotype of these mutants.

### 3.4 Genes involved in flagellar motility

Sixty‐four of the genes we identify in this study resulted in motility impairment as well as biofilm impairment, and they were all identified in the pellicle‐biofilm screening (Table 2). However, only three of the 88 genes required for pellicle‐biofilm formation encoded for proteins directly responsible for the flagellar complex. These were BCE_1756, which encodes a flagellar M‐ring protein, *fliG,* which is the flagellar motor switch protein, and *fliC,* which encodes the assembly protein of the flagellar filament. Flagella are known to be involved in biofilm formation of many different bacterial species, and a properly functioning flagellum requires dozens of genes. In another study, the *fla* and *motAB* genes were shown to be involved in biofilm formation by *B. cereus* (Houry, Briandet, Aymerich, & Gohar, 2010). The remaining genes resulting in nonswimming phenotypes include genes of unknown function, as well as genes of known function which have not been previously reported to effect motility such as *purA* and *lytE*. The relationship between motility and biofilm formation is discussed in more depth below.

### 3.5 Genes involved in DNA replication and/or modification

Gene *purA* encodes an adenylosuccinate synthetase required for purine metabolism. This gene has been previously implicated as important for biofilm formation in *B. cereus* due to what the authors concluded to be a role in the production of extracellular DNA (Vilain, Pretorius, Theron, & Brözel, 2009). Recently, another transposon mutagenesis study identified purine biosynthesis genes *purD* and *purH* to be required for pellicle‐biofilm formation in an environmental isolate of *B. cereus* (Yan et al., 2017). The identification of gene *purA* in our study confirms the importance of purine biosynthesis genes for biofilm formation of *B. cereus*. However, the fact that this mutant was also nonmotile suggests that the presumed lack of extracellular DNA is not soley responsible for its biofilm deficiency.

### 3.6 Genes involved in RNA modification, transcription, and regulation

The largest fraction of transposon insertions (28%) were in the category of RNA modification, transcription, and regulation. Genes in this category include the transcriptional regulator *pdhR* which represses the pyruvate dehydrogenase complex, unknown transcriptional regulators from the LytR, LysR, and MarR families, and the PlcR regulator which has been previously shown to regulate virulence in *B. cereus* (Gohar et al., 2008; Hsueh et al., 2006). It is not surprising to confirm that the PlcR regulator is involved in biofilm formation; however, the three unknown regulators identified in this study represent promising avenues of future research.

Other genes in the category of RNA modification, transcription and regulation include the regulatory protein *yycH*, which belongs to the *yyc*‐operon. The *yycG/yycF* two‐component system contained in this operon is specific to low‐G+C gram‐positive bacteria such as *B. cereus*, and has been shown to positively control autolysin synthesis and biofilm formation in *Staphylococcus aureus* (Dubrac, Boneca, Poupel, & Msadek, 2007). However, the specific role of the regulatory protein YycH in biofilm formation is not known.

### 3.7 Genes involved in sporulation

Two genes involved in sporulation were determined to be necessary for pellicle biofilm formation. Gene *minD* is the septum‐site determining protein (Marston & Errington, 1999; Thomaides, Freeman, El Karoui, & Errington, 2001) located immediately upstream of the choromosome partitioning protein SpoIVFA, which separates the bacterial chromosome prior to sporulation. Gene *jag* is associated with spoIIIJ, a gene which is required for sporulation but is also expressed during vegetative growth (Murakami, Haga, Takeuchi, & Sato, 2002). The link between the dual functions of sporulation and biofilm formation is the global regulatory protein Spo0A, which at intermediate/low levels of phosphorylation turns on genes for biofilm formation and at high phosphorylation levels turns on genes for sporulation (Fujita et al., 2005). The requirement of these genes for biofilm formation may simply be due to an impairment of proper cell division during vegetative growth, resulting in depressed growth rate or sub‐optimal cell morphology for bundling. Alternatively, the Spo0A signaling network may be so profoundly disrupted by the failure of these genes, that the biofilm branch of the Spo0A network is disrupted as well.

### 3.8 Genes for transporters

Six of the transposon insertions (5% of total) resulting in a pellicle‐negative phenotype were in genes encoding transport proteins. Gene BCE_0140 and BCE_02451 encode ABC transporters. Gene BCE_1840 encodes a Na+/H+ antiporter of the NhaC family. Gene BCE_3309 belongs to the major facilitator family of transporters. Gene BCE_3917 encodes a cation‐transporting ATPase, and gene BCE_5200 belongs to the sodium/alanine symporter family of proteins. Transporters might indirectly effect biofilm formation by transporting essential molecules such as nutrients required for total cell fitness, or they might have a more direct effect on biofilm formation by transporting autoinducer molecules or other types of signaling molecules required for biofilm formation (Herzberg, Kaye, Peti, & Wood, 2006). For example, it has been shown that the transition from reversible to irreversible attachment by *Pseudomnas fluorescens* requires an ABC transporter (Hinsa, Espinosa‐Urgel, Ramos, & O'toole, 2003). In what way the six transporters identified here are essential for biofilm formation remains an open avenue of research.

### 3.9 Genes involved in sugar metabolism and exopolysaccharide synthesis

Gene BCE_1975 encodes a deoxyribose phosphate aldolase (*dra* or *deoC)* enzyme, which is involved in the catabolism of deoxynucleosides, deoxyribose, and DNA. This enzyme allows the pentose sugar moiety of these compounds to be used as a carbon and energy source, by shunting it into the glycolysis pathway. Previous studies have shown that adding deoxynucleosides or DNA to the growth media of *B. cereus* induces the expression of *dra* in *B. cereus*, and that the activity of this enzyme increases under anaerobic conditions (Sgarrella et al. 1997). In *B. cereus* 10987, an operon very similar to the *dra‐nupC‐pdp* operon is present in the genome, with the corresponding *deoR* repressor protein located immediately upstream (Figure 1b), as is the case in *B. subtilis* (Saxild, Andersen, & Hammer, 1996). In our study, a transposon insertion in the *dra* gene resulted in impairment of pellicle biofilms, impairment of submerged biofilms, and motility deficiency. This is the first time a link between *dra* and biofilm formation or motility has been shown. We hypothesize that in the tightly packed environment of a *Bacillus* biofilm, cells will experience anaerobic conditions. Forced to abandon respiration for fermentation, these cells will seek to supplement energy yields by channeling deoxyribose 5‐P into glycolysis, which requires a functional *dra* enzyme. How this gene is involved in motility, however, is more enigmatic.

Gene BCE_5588 (*galE*) catalyzes the conversion of galactose to alpha‐D‐glucose. It has recently been shown that galactose metabolism plays a crucial role in biofilm formation by *B. subtilis* (Chai, Beauregard, Vlamakis, Losick, & Kolter, 2012). The appearance of this gene in our screening suggests that galactose metabolism plays a similarly essential role in biofilm formation of *B. cereus*.

*Bacillus cereus* 10987 genome contains a region that has noticeable homology to the *epsABCDEFGHIJKLMNO* operon of *B. subtilis*. A best homologue search using the Integrated Microbial Genome (IMG) platform reveals that the best homologues for *epsB*,* epsC*,* epsF*,* epsL* and *epsN* genes from *B. subtilis* 168 in the *B. cereus* 10987 genome are BCE_5400, BCE_5395, BCE_5390, BCE_5393 and BCE_5394, respectively. All of these genes are located within a 21‐gene region spanning genes BCE_5401 to BCE_5380. This genomic region has the highest degree of sequence homology to the *B. subtilis eps*‐operon of any region in the *B. cereus* 10987 genome. Four of the genes we identified in this study fall into this *eps*‐like region, and they are all genes involved in sugar metabolism. Transposon insertions in genes BCE_5356 (a putative glycosyl transferase domain protein), BCE_5385 (a UDP‐glucose 6‐dehydrogenase), gene BCE_5386 (a putative polysaccharide transport protein) and gene BCE_5398 (a capsular exopolysaccharide family protein) all resulted in a biofilm‐negative phenotype. Based on these observations, it would be reasonable to hypothesize that this genomic region plays a role in the production of biofilm matrix exopolysaccharides. Precisely this genomic region was knocked out by Gao and colleagues in *B. cereus* strain 905, in order to observe the effect on biofilm formation. Disrupting this gene region in strain 905 had only a minimal effect on pellicle biofilm formation (Gao, Foulston, Chai, Wang, & Losick, 2015), and this *eps*‐like region has henceforth been considered to be unnecessary for biofilm formation in *B. cereus* (Majed, Faille, Kallassy, & Gohar, 2016). In our study, however, the transposon insertions in this *eps*‐homologous region resulted in a total inability to form a pellicle biofilm after 3 days of incubation (the same time frame as the study by Gao et al.). This suggests that the importance of this *eps*‐like region for biofilm formation in *B. cereus* is strain‐specific, or perhaps dependent on the specific growth conditions.

Our study also identified a second region which could be involved in the production of an exopolysaccharide component of the biofilm matrix. Three of our biofilm‐negative mutants harbored transposon insertions in three genes that lie within the same operon; BCE_5583‐5587. BCE_5585, BCE_5586, and BCE_5587 fall into a cluster of five consecutive genes, located so closely together that their coding sequences somewhat overlap. Transposon insertions in genes BCE_5585 and BCE_5587 resulted in impairment of biofilm formation in both screening assays, while gene BCE_5586 was identified only in the pellicle screening.

Genes BCE_5583‐5587 are annotated in GenBank as putative proteins of unknown function, with the exception of BCE_5586 which is annotated as a putative glycosyl transferase. These enzymes transfer saccharide moieties from an activated nucleotide sugar to an acceptor molecule. Based on protein domain analysis by Pfam, BCE_5587 contains a PelG‐domain, which in *Pseudomonas aeruginosa* identifies exopolysaccharide biosynthesis. On the bases of sequence homology we therefore propose that operon BCE_5583‐5587 could be involved in the synthesis of an extracellular polysaccharide, necessary for the proper formation of the biofilm matrix in this strain of *B. cereus*. The function of this novel biofilm‐associated operon remains to be experimentally determined, and we can therefore only hypothesize as to its function.

The *B. cereus* strain we used in our study has been determined to have a higher carbohydrate:protein ratio than the *B. cereus* type strain ATCC 14579 (Karunakaran & Biggs, 2010), which suggests that extracellular polysaccharides could be at least partially responsible for the superior biofilm forming ability of this strain. Interestingly, while strain ATCC 10987 contains the BCE_5583‐5587 operon which we propose is involved in polysaccharide‐matrix production, the *B. cereus* type strain ATCC 14579 genome does not contain this operon. Of all the complete genome sequences of *B. cereus* strain currently available in the JGI Integrated Microbial Genomics database, approximately 30% contain the complete BCE_5583‐5587 operon to 80% identity.

In our study, transposon insertions in both the *eps*‐like region, and operon BCE_5583‐5587, caused a biofilm‐negative phenotype, although only insertions in the EPS‐like operon impaired motility. The two operons are therefore both essential for biofilm formation, but presumably do so via different, yet complementary, mechanisms. The presence of one operon was not sufficient to maintain biofilm formation, if the other operon was disrupted. This suggests that the two operons work together to achieve their function, possibly by encoding unique polysaccharides. Both are necessary, but not sufficient, for biofilm formation.

### 3.10 Other genes required for biofilm formation

Nine of the identified genes did not fall into any of the above categories. One of these genes, BCE_0696, is annotated in GenBank as a “sensory box/GGDEF family protein,” and is an orthologue of the diguanylate cyclase gene *cdgF*. The diguanylate cyclase enzymes produce cyclic‐di‐GMP from two molecules of GTP, and are characterized by the presence of a GGDEF domain (Hickman, Tifrea, & Harwood, 2005; Paul et al., 2004; Ryjenkov, Tarutina, Moskvin, & Gomelsky, 2005). Cyclic‐di‐GMP is known to be a ubiquitous biofilm signaling molecule throughout the bacterial domain (Bobrov et al., 2006; Jenal & Malone, 2006; Waters, Lu, Rabinowitz, & Bassler, 2008), and is also important for biofilm formation in *B. subtilis* (Chen, Chai, Guo, & Losick, 2012). A recent investigation into the role of cyclic‐di‐GMP in biofilm formation in the *B. cereus* group of organisms was performed by Fagerlund et al. (2014), in which GGDEF‐domain containing genes from the *B. cereus* group of organisms were expressed in *Bacillus thuringiensis*. Deletion of gene *cdgF* (BC_0628) not only completely abolished biofilm formation in microtiter biofilm formation assays, but was the only deletion mutant in the library in which biofilm formation was completely abolished. The authors confirmed the role of this gene as a diguanylate cyclase (Fagerlund et al., 2014). In our study, two independent transposon insertions into *cdgF* (BCE_0696) resulted in deficiencies in biofilm formation (both pellicle and submerged) and motility, thereby demonstrating *in situ* that *cdgF* is required for biofilm formation in *B. cereus* ATCC 10987.

Gene BCE_3314 is a putative member of the cholesterol‐dependent family of cytolysins (previously known as thiol‐activated cytolysins). These toxins disrupt cell membranes by forming large pores up to 30 nm in diameter. This cytolytic activity specifically requires the presence of cholesterol in membranes but does not depend on any other specific cell‐surface receptor (Palmer, 2001). Cytolysins of this family are able to lyse the cytoplasmic membranes of almost all animal cells and therefore play an important role in virulence. Why this gene is required for biofilm formation is a fascinating question. Cytolytic syphgomylinases have been shown to promote biofilm formation in *S. aureus* by binding to extracellular DNA and forming an insoluable complex (Huseby et al., 2010). Cytolytic enzymes may therefore fill multiple roles, with biofilm‐promoting mechanisms potentially very different from the primary cytolytic functions of the enzymes.

### 3.11 The relationship between biofilm formation and motility

Although the majority of disrupted genes resulting in a pellicle‐negative phenotype are not known to be directly involved in motility, we observed that the majority of the 88 mutants were also motility impaired. Of the 70 mutants tested for the ability to swim in a soft‐agar swimming assay, 63 were unable to swim. We therefore conclude that the ability to swim is a pivotal factor in the formation of a pellicle biofilm by *B. cereus* 10987. This observation is in agreement with a study by Houry and colleagues, who concluded that motility is necessary for biofilm formation by *B. cereus* strain 407 in static assays where functional flagella aid the bacteria in reaching the air‐water interface (Houry et al., 2010). In our screening for mutants unable to form a submerged biofilm on the surface of a peg suspended in nutrient broth, five of the seven identified mutants were unable to swim (Table 3). These mutants were presumably able to contact the submerged surface by chance, during the process of sedimentation, while the motile mutants were able to contact the surface of the peg by swimming to it, and their impairment in biofilm formation occurred at a later stage of biofilm development. The two motile mutants (BCE_5585 and BCE_5587), have transposon insertions in genes belonging to operon BCE_5583‐5587 which we hypothesize is required for the production of exopolysaccharide matrix components.

With the exception of three genes required for flagellar assembly, the other swimming‐deficient mutants retain functional copies of all genes required for motility and chemotaxis. Motility deficiency in these mutants must therefore be a secondary effect of the transposon insertion, effecting either motility or chemotaxis pathways in ways that are not yet understood.

**Figure 3:**
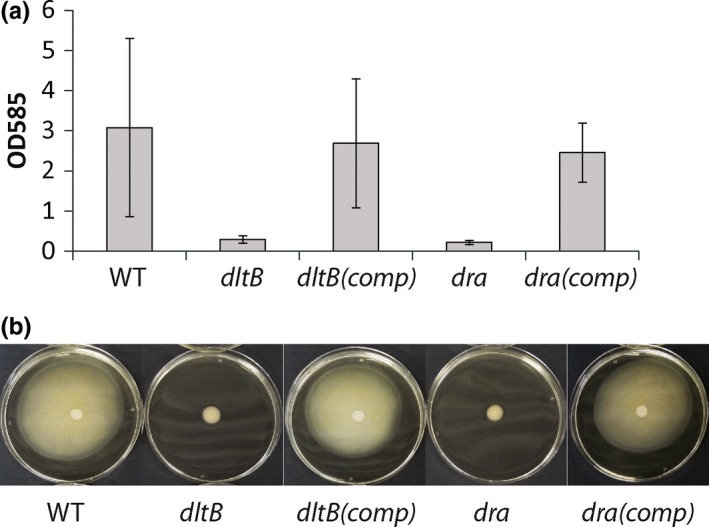
Genetic complementation of *dlt*‐ and *dra*‐operons restores a) biofilm‐forming and b) swimming phenotypes of mutants with transposon insertions in genes *dltB* and *dra*

We found it puzzling that many mutants, harboring transposon insertions in genes with no clear connection to motility, were nevertheless unable to swim. In order to rule out the possibility that secondary mutations unrelated to the transposon insertion were responsible for swimming‐ and biofilm‐impairment, we selected mutants with insertions in genes *dra* and *dltB* for genetic complementation. The entire *dra‐nupC‐pyp* operon and the entire *dlt*‐operon were PCR amplified from the wildtype genome using primers shown in Table 1 (amplified regions are indicated by grey bars in Figure 1b). These amplicons were ligated into shuttle vector pMK3. pMK3‐*dra* and pMK3‐*dlt* were then transformed into the transposon mutants corresponding to these genes. This complementation was, in both cases, sufficient to restore swimming and biofilm phenotypes to wild type levels (Figure 3), which confirms that the mutant phenotypes were caused solely by transposon insertions in these genes. We are therefore able to demonstrate, for the first time, that the *dlt*‐ and *dra*‐operons are required for swimming motility in *B. cereus*. This observation may partially explain the observation by Khattar et al. (2009) that the *dlt*‐operon is required for virulence in *B. cereus*, as mutants which are motility deficient will not be able to spread within a host.

### 3.12 Screening assays for pellicle and submerged biofilms did not produce the same result

Due to the structural uniqueness of the two types of *B. cereus* biofilms, we expected to identify different genes in our screening assays for pellicle and submerged‐biofilm formation. However, only three of six genes identified to be necessary for submerged‐biofilm formation were unique to this assay and not identified in the pellicle‐biofilm assay. The reason for this lies partially in the small number of genes identified as necessary for submerged‐biofilm formation. The submerged‐biofilm assay was much more conservative in determining biofilm‐negative mutants. This is due to the way *B. cereus* forms a biofilm in the peg‐lid biofilm assay. *B. cereus* cells attach strongly around the peg‐lid, predominantly at the three‐way solid‐liquid‐air interface. A ring is formed around the peg at the level of the air‐liquid interface, which is the major surface‐attachment point for the biofilm. Cells spread out from this attachment point across the surface of the media. Although the biofilm is attached to the peg‐lid, this attachment site is minimal, and majority of biomass can be easily washed away during the multiple washing steps of the crystal violet assay. This assay is therefore an extremely stringent screening method for biofilm formation, identifying only those mutants which are so heavily impaired in biofilm formation that they resemble the bacteria‐free negative control. Despite the small number of mutants identified, interesting conclusions can be drawn from the results. Two of the three genes identified to be submerged‐biofilm negative but not identified in the pellicle screening (BCE_5585 and BCE_5587) belong to the novel operon we propose to be involved in production of the biofilm polysaccharide matrix (BCE_5583‐5587). They were not identified in the pellicle screening possibly because they retain their ability to swim (Table 3) and attach to a surface, though the development of a mature biofilm is impaired. These results provide further evidence that the pellicle‐screening assay selects heavily for mutants deficient in swimming.

### 3.13 Comparison of results to other studies

A recent study identified 23 genes required for pellicle‐biofilm formation in an environmental isolate of *B. cereus* (Yan et al., 2017). The authors reported the identities of these genes as the corresponding locus tags in *B. cereus* ATCC 14579. To compare these results with our own, we identified the best homologue of these 23 genes in *B. cereus* ATCC 10987. We were therefore able to determine that there is no direct overlap between the 23 genes they identified in their study, and the 91 genes we identified in ours. This lack of similarity may be due to the different methods used to identify those genes. Yan and colleagues first identified unusual colony morphologies before testing for pellicle biofilm defects, while we screened directly for pellicle deficiencies. Which genes are important for biofilm formation may also be, to some extent, strain dependent. Another possible reason for the lack of overlap between results is the relatively small number of genes identified in both studies. However, some broad similarities can be identified by comparing these independently produced sets of results. Both sets of genes include *pur* genes, which implicate purine biosynthesis as essential for biofilm formation. The relationship between *pur* genes and biofilm formation has previously been investigated in terms of the role of extracellular DNA in biofilm formation (Vilain et al., 2009). Also, both our study and that by Yan et al. identified genes required for sporulation. Although the genetic basis of sporulation and biofilm formation are known to be intimately linked via the Spo0A transcriptional regulon, sporulation is generally thought of as a process that occurs after biofilm formation has been established (when phosphorylation of Spo0A has increased past the low, biofilm‐inducing levels), and it is therefore curious that *B. cereus* is unable to form a biofilm when sporulation genes are disrupted.

In *B. cereus*, or closely related species, key genes for biofilm formation have been identified, such as the *sinR* transcriptional regulator in *B. thuringiensis* (Fagerlund et al., 2014), *sipW* or *tasA* (Caro‐Astorga et al., 2015) and the *codY* pleiotrophic regulator (Lindbäck et al., 2012). None of these genes, however, were identified in our study. Rather than indicating that these genes are unimportant, the fact that we did not identify these genes is most likely due to an incomplete identification of genes required for biofilm formation. A library of 5019 transposon mutants does not cover the 5603 genes annotated by GenBank in the *B. cereus* 10987 genome, and we therefore did not achieve a global coverage of the genome.

## 4 CONCLUSIONS

This study identifies 91 genes required for biofilm formation by *B. cereus*. Among these genes are several which have not previously been linked to biofilm formation, such as *dra* and the BCE_5583‐5587 operon. Many genes, such as diguanyate cyclase (BCE_0696), *gidAB* and *dltB*, are known from biofilm formation in other species, and this study has confirmed that they are also essential for biofilm formation in *B. cereus*. In *B. subtilis*, it has been shown that galactose metabolism plays a crucial role in biofilm formation (Chai et al., 2012). Our identification of gene *galE* as essential for biofilm formation in *B. cereus* suggests that galactose metabolism plays a similarly important role in biofilm formation by *B. cereus*. The majority of genes identified in this study impaired not only biofilm formation, but also motility. This means that the effect on biofilm formation may be an indirect one, resulting from the inability of the cells to reach the surface.

Three transcriptional regulators belonging to the LytR, LysR, and MarR families, as well as six transporters identified here represent promising avenues of future research. Considering that the exact function of these genes is unknown, how they influence biofilm formation is a fascinating unresolved question. One of our transposon mutants (*speD*) suggests that spermine and spermidine may be necessary for biofilm formation in *B. cereus*, which is a link that has not yet been proven experimentally. Additionally, gene BCE_3314 is homologous to a member of the cholesterol‐dependent family of cytolysins, raising questions about the relationship between cytolysis and the ability to form a biofilm.

A major gap in our current understanding of biofilm formation by *B. cereus* is the identification of the genes encoding the polysaccharide component of the biofilm matrix (Majed et al., 2016). Our study has identified two genomic regions required for biofilm formation which may be involved in the production of the polysaccharide components of the biofilm matrix. One of these regions (the *eps*‐like operon) has been previously discounted as important for biofilm formation due to one study of a single strain. The importance of the *eps*‐like operon in *B. cereus* is very likely strain‐dependent, and deserves further investigation in a larger selection of *B. cereus* strains. The second genomic region possibly contributing to polysaccharide matrix production is a novel operon of unknown function (BCE_5583‐5587). We hypothesis that this operon plays an important role in matrix polysaccharide production in the strains in which it is present. The discovery of novel genes required for biofilm formation, including an unknown operon, demonstrates that the genetic basis of biofilm formation by *B. cereus* is not yet fully elucidated. Further molecular investigations coupled with *in vitro* experimentation are necessary to fully uncover the genetic regulation of biofilm formation in *B. cereus*.

## CONFLICT OF INTEREST

None declared.
