# Supplementary material for: A transposon mutant library of Bacillus cereus ATCC 10987 reveals novel genes required for biofilm formation and implicates motility as an important factor for pellicle‐biofilm formation
Source: Microbiologyopen. 2017 Nov 22;7(2):e00552. doi: 10.1002/mbo3.552 (PMC5911993; doi:10.1002/mbo3.552)
Supplement: Supplementary file 1 [file MBO3-7-na-s001.docx]

**Table S1**: Genes identified in this study to be essential for pellicle-biofilm formation by *B. cereus*

| **Gene Locus tag** | **Number of independent transposon insertions** | **Location of transposon insertion (base pair number)** | **Gene name / annotation** | **Predicted function** | **Pathway/Functional group** | **swim (yes/no)** |
| --- | --- | --- | --- | --- | --- | --- |
| **AMINO ACID METABOLISM** | | | | | | |
| BCE_0585 | 1 | 594681 | *gltB* | glutamate synthase, large subunit | alanine, aspartate and glutamate metabolism; biosynthesis of amino acids | no |
| BCE_0688 (upstream) | 1 | 708483 | *kbl* | glycine C-acetyltransferase | Glycine, serine and threonine metabolism | no |
| BCE_1486 | 2 | 1475690; 1476433 | *dltB* | membrane protein involved in export of D-alanine | cationic antimicrobial peptide (CAMP) resistance; two component systems; | N/A |
| BCE_1487 | 2 | 1477422; 1,477,539 | *dltA* | D-alanine activating enzyme / D-alanine poly(phosphoribitol) ligase subunit | D-Alanine metabolism; cationic antimicrobial peptide (CAMP) resistance; two component systems | no |
| BCE_4009 | 2 | 3755911, 3756305 | *cadA* | lysine decarboxylase | Lysine degradation (amine acid metabolism); tropane, piperidine and pyridine alkaloid biosynthesis (biosynthesis of secondary metabolities) | no |
| BCE_4209 (upstream) | 1 | 3925781 | *sdhB* | L-serine dehydratase, iron-sulfur dependant, beta subunit | Glycine, serine and threonine metabolism; cysteine and methionine metabolism; biosynthesis of amino acids | yes |
| BCE_4454 | 1 | 4126277 | *mccB* | cystathionine beta-lyase / homocysteine desulfhydrase | Glycine, serine and threonine metabolism; cysteine and methionine metabolism; sulfur metabolism; biosynthesis of amino acids | no |
| BCE_4712 | 1 | 4365352 | *speD* | s-andenosylmethionine decarboxylase proenzyme; catalyzes the decarboxylation of S-adenosylmethionine to S-adenosylmethioninamine | Cystein and methionine metabolism; Arginine and proline metabolism; amino acid metabolism (Methionine salvage pathway; Polyamide biosynthesis, arginine -> agmatine -> putriscine -> spermidine | no |
| **CELL GROWTH AND DIVISION** | | | | | | |
| BCE_5742 | 1 | 32104 | Bc23SB | 23S ribosomal RNA | ribosome biogenesis | no |
| BCE_5745 | 1 | 84328 | Bc23SC | 23S ribosomal RNA | ribosome biogenesis | no |
| BCE_0097 (upstream) | 1 | 104890 | ribosomal protein L11 |  | ribosome biogenesis | no |
| BCE_5763 | 1 | 335253 | Bc23S1 | 23S ribosomal RNA | ribosome biogenesis | yes |
| BCE_1984 | 1 | 1917753 | *topB* | DNA topoisomerase III | DNA repair and recombination / DNA separation during cell division | no |
| BCE_3964 | 1 | 3717614 | *rsmH* (*mraW*) | 16S rRNA methyltransferase | ribosome biogenesis | N/A |
| BCE_4125 | 1 | 3857285 | ScpA | segregation and condensation protein A | chromosome partitioning protein | no |
| BCE_4353 | 1 | 4038796 | cell elongation-specific peptidoglycan D,D-transpeptidase |  | cell elongation | no |
| BCE_4561 | 2 | 4232212; 4232427 | Ion | ATP-dependant protease | Cell cycle - Caulobacter (cell growth and death) | no |
| BCE_5301 | 1 | 4887884 | *lytE* | endopeptidase; cell wall hydrolyses | cell division | no |
| BCE_5423 | 1 | 5009075 | *nuoC* | NADH dehydrogenase I, C subunit | oxidative phosphorylation | no |
| BCE_5633 | 1 | 5218114 | *gidB* | 16S rRNA (guanine527-N7)-methyltransferase | ribosome biogenesis/modification | N/A |
| BCE_5634 | 2 | 5219259; 5,219,518 | *gidA* | tRNA uridine 5-carboxymethylaminomethyl modification enzyme | chromosome partitioning | no |
| **FLAGELLAR MOTILITY** | | | | | | |
| BCE_1756 | 1 | 1710069 | flagellar M-ring protein |  | Flagellar assembly / bacterial chemotaxis | no |
| BCE_1767 | 1 | 1710735 | *fliG* | flagellar motor switch protein | Flagellar assembly / bacterial chemotaxis | no |
| BCE_1780 | 2 | 1722347; 1722507 | *fliC* | flagellin | Flagellar assembly / bacterial chemotaxis | no |
| **DNA REPLICATION/MODIFICATION** | | | | | | |
| BCE_1692 | 1 | 1646268 | *dinG* | ATP-dependant DNA helicase | DNA repair and recombination | N/A |
| BCE_5617 | 1 | 5204841 | *purA* | Adenylosuccinate synthetase; adenine ribonucleotide biosynthesis | purine metabolism; alanine, aspartate and glutamate metabolism | no |
| BCE_A0091 | 1 | plasmid88,273 | *dnaN* | DNA polymerase | purine metabolism; pyrimidine metabolism; DNA replication; mismatch repair | N/A |
| **RNA MODIFICATION/TRANSCRIPTION/REGULATION** | | | | | | |
| BCE_0051 | 13 | 54778; 55,006; 55,088; 56,162;56,515; 56,970; 57,049; 57,202; 57,229; 57,624; 57,733; 57,799 | *mfd* | transcription-repair coupling factor (superfamily II helicase), does nucleotide excision repair. | transcription repair | N/A |
| BCE_0542 | 1 | 554874 | RNA methyltransferase, TrmA family |  | Transfer RNA biogenesis | no |
| BCE_0875 | 3 | 901,507; 901,619; 901,882 | transcription antiterminator, LytR family |  | transcriptional regulator | no |
| BCE_1415 | 1 | 1418392 | *pdhR* | transcriptional regulator, GntR family, represses pyruvate dehydrogenase complex | transcriptional regulator | no |
| BCE_3833 | 1 | 3583282 | rnj | riboneuclease J | RNA degradation | no |
| BCE_4026 | 4 | 3768001; 3768457; 3768588; 3768591 | RNase J1 | ribonuclease J | RNA degradation | no |
| BCE_4150 | 2 | 3874856; 3875001 | transcriptional regulator marR family |  | transcriptional regulator | no |
| BCE_4392 (upstream) | 1 | 4073791 | RsmE | 16S rRNA(uracil1498-N3)-methyltransferase | 16S rRNA modification factor | no |
| BCE_4687 (upstream) | 1 | 4345490 | *pheST* | phenylalanyl-tRNA synthetase, alpha and beta subunits | Aminoacyl tRNA biosynthesis | yes |
| BCE_5280 | 1 | 4870304 | transcriptional regulator, LysR family |  | transcriptional regulator | N/A |
| BCE_5481 | 2 | 5060626; 5060653 | plcR, putative | transcriptional regulator | transcriptional regulator | N/A |
| BCE_5613 | 1 | 5200504 | YycH | regulatory protein | Regulation/signal transduction | no |
| BCE_5635 | 1 | 5220872 | *trmE* | tRNA modification methyltransferase GTPase | transcription | N/A |
| **SPORULATION** | | | | | | |
| BCE_4540 | 1 | 4208761 | *minD* | septum-site determining protein MinD | sporulation related gene, comes right before spoIVFA, chromosome partitioning protein, inhibitor of FTSZ assembly | no |
| BCE_5636 | 1 | 5222600 | *jag* | spoIIIJ-associated protein | sporulation | no |
| **SUGAR METABOLISM** | | | | | | |
| BCE_1975 | 1 | 1909330 | *deoC*/*dra* | deoxyribose phosphate aldolase | pentose phosphate pathway | no |
| BCE_5356 | 1 | 4943951; 4943954 | glycosyl transferase domain protein, putative |  | carbohydrate/sugar metabolism | no |
| BCE_5385 | 1 | 4972962 | *ugd* | UDP-glucose 6-dehydrogenase | starch and sucrose metabolism; (ascorbate biosynthesis, glucose-1P -> ascorbate; Glucuronate pathway (uronate pathway); Nucleotide sugar biosynthesis | no |
| BCE_5386 | 1 | 4974317 | polysaccharide transport protein, putative |  | polysaccharide transporter | no |
| BCE_5398 | 1 | 4987306 | capsular exopolysacchraide family protein |  | exopolysaccharide production | no |
| BCE_5586 | 1 | 5175279 | glycosyl transferase, group 1 family protein |  | carbohydrate/sugar metabolism | N/A |
| BCE_5588 (downstream) | 1 | 5177285 | *galE* | catalizes conversion of galactose to alpha-D-glucose | Galactose metabolism; amino sugar and nucleotide sugar metabolism (nucleotide sugar biosynthesis; galactose degradation, ) | no |
| **TRANSPORTERS** | | | | | | |
| BCE_0140 | 1 | 136978 | ABC transporter, ATP binding protein |  | transporter | no |
| BCE_0245 | 1 | 247451 | ABC transporter, ATP-binding protein |  | transporter | no |
| BCE_1840 | 1 | 1777050 | Na+/H+ antiporter, NhaC family |  | transporter | N/A |
| BCE_3309 | 1 | 3099433 | major facilitator family transporter |  | transporter | no |
| BCE_3917 | 1 | 3665382 | cation-transporting ATPase, E1-E2 family |  | transporter | no |
| BCE_5200 | 1 | 4795500 | sodium/alanine symporter family protein |  | transporter | no |
| **OTHER** | | | | | | |
| BCE_0194 | 1 | 202242 | *fabG* | 3-oxoacyl-[acyl-carrier protein] reductase (fatty acid biosynthesis) | Fatty acid biosynthesis, biotin metabolism, biosynthesis of unsaturated fatty acids, fatty acid metabolism | no |
| BCE_0433 | 1 | 446520 | mandelate racemase/muconate lactonizing enzyme family protein |  | aromatic acid catabolism | N/A |
| BCE_0722 (upstream) | 1 | 745779 | universal stress protein family |  | stress response | N/A |
| BCE_1423 (upstream) | 1 | 1425517 | potassium uptake protein, TrkH family |  | potassium uptake | N/A |
| BCE_5120 | 1 | 4727872 | *sufC* | Fe-S cluster assembly/ ATP-binding protein | Assembly of Fe-S culters | no |
| BCE_0696 | 1 | 718076 | sensory box/GGDEF family protein | diguanylate cyclase; converts two GTP to cyclic di-GMP | nucleotide metabolism; virulence signaling | no |
| BCE_1125 | 1 | 1131871 | *glpK* | glycerol kinase | gycerolipid metabolism, PPAR signaling pathway, plant-pathogen interaction | N/A |
| BCE_3314 | 1 | 3104049 | *slo* | thiol-activated cytolysin | Quorum sensing; Nod-like receptor signaling pathway | no |
| BCE_5571 | 1 | 5158425 | antiholin-like protein LrgB |  | two-componant system (signal transduction) | no |
| **UNKNOWN** | | | | | | |
| BCE_0092 | 1 | 102685 | conserved hypothetical protein | (yacP-like; potential RNase due to NYN domain)(Ananthataman 2006) | unknown | N/A |
| BCE_0529 | 1 | 543808 | hypothetical protein |  | unknown | no |
| BCE_0687 (upstream) | 1 | 708483 | hypothetical protein |  | unknown | no |
| BCE_0752 | 2 | 777571; 777,799 | conserved hypothetical protein |  | unknown | no |
| BCE_0766 | 1 | 791589 | acetyltransferase, GNAT family |  | unknown | no |
| BCE_1138 | 1 | 1143966 | hypothetical protein |  | unknown | yes |
| BCE_1161 | 1 | 1171530 | lipoprotein, putative |  | unknown | N/A |
| BCE_1503 | 1 | 1486560 | conserved hypothetical protein |  | unknown | yes |
| BCE_1736 | 1 | 1685321 | NAD(P)H-flavin oxidoreductase |  | unknown | no |
| BCE_2473 (preceding) | 1 | 2357814 | conserved hypothetical protein |  | unknown | no |
| BCE_2700 | 1 | 2571170 | hypothetical protein |  | unknown | no |
| BCE_3189 | 1 | 2983010 | conserved hypothetical protein |  | unknown | no |
| BCE_3332 | 1 | 3120527 | hypothetical protein |  | unknown | N/A |
| BCE_3735 | 1 | 3484667 | membrane protein, putative |  | unknown | no |
| BCE_3739 | 1 | 3487942 | conserved hypothetical protein |  | unknown | no |
| BCE_4837 (preceding) | 1 | 4469695 | conserved hypothetical protein |  | unknown | no |
| BCE_5182 | 1 | 4781058 | conserved hypothetical protein |  | unknown | no |
| BCE_5304 | 1 | 4890209 | degV family protein |  | unknown | no |
| BCE_5361 | 1 | 4949640 | conserved hypothetical protein |  | unknown | no |
| BCE_5362 | 1 | 4949640 | conserved hypothetical protein |  | unknown | no |
| preceeding BCE_5439 | 1 | 5023948 | hypothetical protein |  | unknown | yes |
| BCE_5499 | 1 | 5087177 | hypothetical protein |  | unknown | N/A |
| BCE_5587 | 1 | 5176526 | transmembrane protein, ortholog TM1408, putative |  | unknown | yes |
| BCE_5591 | 2 | 5179626; 5,179,788 | conserved hypothetical protein |  | unknown | no |
| BCE_A0124 | 1 | plasmid116,024 | conserved hypothetical protein |  | unknown | no |
